# The development of anticyclic citrullinated peptide (anti‐CCP) antibody following severe COVID‐19

**DOI:** 10.1002/iid3.1276

**Published:** 2024-05-23

**Authors:** Seyed Askar Roghani, Mohammad Dastbaz, Ramin Lotfi, Afsaneh Shamsi, Zahra Abdan, Rezvan Rostampour, Bijan Soleymani, Mohammad Hossein Zamanian, Parviz Soufivand, Mehran Pournazari, Mahdi Taghadosi

**Affiliations:** ^1^ Immunology Department, Faculty of Medicine Kermanshah University of Medical Sciences Kermanshah Iran; ^2^ Clinical Research Development Center, Imam Reza Hospital Kermanshah University of Medical Sciences Kermanshah Iran; ^3^ Medical Biology Research Center, Health Technology Institute Kermanshah University of Medical Sciences Kermanshah Iran; ^4^ Blood Transfusion Research Center, High Institute for Research and Education in Transfusion Medicine Kurdistan Regional Blood Transfusion Center Sanandaj Iran; ^5^ Clinical Research Development Center, Tohid Hospital Kurdistan University of Medical Sciences Sanandaj Iran; ^6^ Department of Clinical Biochemistry, Medical School Kermanshah University of Medical Sciences Kermanshah Iran

**Keywords:** anti‐CCP, autoantibodies (AABs), autoimmunity, COVID‐19, SARS‐CoV‐2

## Abstract

**Objectives:**

The dysregulated immune response is one of the cardinal features of severe coronavirus disease 2019 (COVID‐19). This study was conducted to clarify the occurrence of autoantibodies (AABs) associated with systemic autoimmune rheumatic diseases (SARDs) in hospitalized patients with a moderate, severe, and critical form of COVID‐19.

**Methods:**

The serum samples obtained from 176 hospitalized COVID‐19 patients were investigated in this study, including patients with moderate (*N* = 90), severe (*N* = 50), and critical (*N* = 36) forms of COVID‐19. Also, the serum samples collected from healthy subjects before the COVID‐19 pandemic were used as controls (*N* = 176). The antinuclear antibodies (ANAs), antidouble‐stranded DNA (anti‐dsDNA), cytoplasmic‐anti neutrophil cytoplasmic antibody (c‐ANCA), perinuclear ANCA (p‐ANCA), antiphospholipid antibodies (aPLs), and anticyclic citrullinated peptide (anti‐CCP) occurrence was evaluated using a solid‐phase enzyme‐linked immunosorbent assay (ELISA).

**Results:**

The results showed that the occurrence of ANAs, anti‐dsDNA, anti‐CCP, c‐ANCA, and p‐ANCA was significantly higher in the COVID‐19 patients compared to serum obtained from healthy subjects (*p* < .0001, *p* < .0001, *p* < .0001, *p* < .05, and *p* < .001, respectively). The positive number of anti‐CCP tests increased significantly in severe COVID‐19 compared to the moderate group (*p* < .01).

**Conclusion:**

Our study further supports the development of autoantibodies related to systemic autoimmune rheumatologic diseases. To the best of our knowledge, this is the first study with a large sample size that reported the occurrence of anti‐CCP in a severe form of COVID‐19.

## 1 INTRODUCTION

Autoantibodies (AABs) have an unprecedented role in the initiation, progression, and severity of systemic autoimmune rheumatic diseases (SARDs) and serve as prognostic and diagnostic tools in autoimmune disease management.^1^ Some antibodies like anticyclic citrullinated peptide (anti‐CCP) in rheumatoid arthritis (RA) become positive in the serum a median of 4.5 years before the initiation of clinical manifestations.^2^ Viruses are among the most acknowledged environmental factor that elicits the production of AABs in systemic and organ‐specific autoimmune disorders.^3^

Coronavirus disease 2019 (COVID‐19) has infected many individuals globally, with heterogeneous manifestations from mild to severe and critical clinical outcomes.^4^, ^5^, ^6^ Severe acute respiratory syndrome‐coronavirus 2 (SARS‐CoV‐2), the viral agent of COVID‐19, elicits the production of AABs through several mechanisms, including toll‐like receptor (TLR) engagement, eliciting extrafollicular B cell response, cytokine storm, and neutrophil extracellular traps (NETs) formation.^3^, ^6^, ^7^ Previous investigations, including studies conducted by Woodruff et al.^7^ and Anaya et al.,^8^ showed the production of AABs associated with rheumatologic disorders, especially systemic lupus erythematosus (SLE) in COVID‐19, which was reviewed comprehensively by Taghadosi et al.^3^ Among several AABs reported in COVID‐19 patients, the AABs associated with systemic autoimmune disorders, including antinuclear antibodies (ANAs) and antiphospholipid antibodies (aPLs), have an association with some clinical manifestations of COVID‐19, including long‐COVID, thrombosis, and venous occlusion.^9^, ^10^, ^11^, ^12^

The extent of antibody production in COVID‐19 is enormous and the presence of COVID‐19‐specific AABs has implications in stratifying patients into specific disease phenotypes. In this context, rheumatoid factors (RFs) specific to COVID‐19 have been reported in the study of Amjadi et al.^13^ To further clarify the development of AABs associated with systemic autoimmune disorders in COVID‐19 hospitalized patients, in the current investigation, we assessed the occurrence of AABs reported in rheumatological diseases, including AABs developed in antineutrophil cytoplasmic antibody (ANCA)‐associated vasculitis (AAV), rheumatoid arthritis (RA), systemic lupus erythematosus (SLE), and antiphospholipid syndrome (APS). Some AABs, including anti‐CCP, became positive several years before the development of RA in the preclinical phase.^14^ Finding the occurrence of these prognostic AABs in COVID‐19‐affected populations may predict the increased risk for the incidence of autoimmunity and systemic autoimmune diseases in the future. Although using single‐cell B cell repertoire analysis, Woodruff et al.^7^ documented the presence of autoreactive antibodies associated with rheumatological disorders; the number of COVID‐19 patients evaluated in this study was small. In a similar study, Anaya et al.^8^ documented the presence of rheumatic and phospholipid autoantibodies in a larger number of COVID‐19 patients (*n* = 120). Regarding the complex effect of population genetic and environmental factors in the development of autoimmune response and the differences in the prevalence of autoimmunity in various populations, the present study aimed to further clarify the occurrence of AABs associated with systemic autoimmune rheumatic diseases (SARDs) in hospitalized patients with a moderate, severe, and critical form of COVID‐19 with larger sample volume (*n* = 176) in Kermanshah province, which is located in the west of Iran.

## 2 MATERIALS AND METHODS

### 2.1 Study design and participants characteristics

In this study, blood samples were taken from 176 patients with COVID‐19 (M/F: 81/95) after 2 weeks of hospitalization in Golestan Hospital, Kermanshah, Iran. The COVID‐19 infection was confirmed by the real‐time reverse transcriptase‐polymerase chain reaction (RT‐PCR) technique. Based on COVID‐19 severity, patients were stratified into three groups: moderate (*N* = 90), severe (*N* = 50), and critical (*N* = 36), according to clinical and laboratory parameters assessed by a specialist physician. In brief, patients who showed evidence of lower respiratory illness during clinical assessment or imaging and who had an oxygen saturation measured by pulse oximetry (SpO_2_) ≥94% on room air at sea level were considered as moderate. Moreover, patients who had a SpO_2_ < 94% on room air at sea level, a ratio of arterial partial pressure of oxygen to fraction of inspired oxygen (PaO_2_/FiO_2_) < 300 mmHg, a respiratory rate >30 breaths/min, or lung infiltrates >50% were regarded as severe. Noteworthy, patients who possessed respiratory failure, septic shock, or multiple organ dysfunction were considered as critical. Besides, for the control samples, we used 176 serum samples from sex‐ and age‐matched healthy subjects before November 2019, lacking any background of autoimmune diseases. The demographic and clinical features of the studied groups are shown in Table 1. This study was in accordance with the Declaration of Helsinki and was approved by the Ethics Committee of the Kermanshah University of Medical Sciences (Ethical code: IR.KUMS.MED.REC.1400.058). The procedure of the study was clarified for all participants, and they were informed about the aims of the research and then signed the informed consent form.

**Table 1:** Clinical characteristics of groups (healthy controls and patients group who categorized into three subgroups namely moderate, severe, and critical) and also the number of positive tests based on manufacturers cut off.

Variables	Moderate	Severe	Critical	*p* Value	Patients	Controls	*p* Value	Cut off (U/mL)
*N*	90	50	36		176	176		
Age (mean ± SEM)	55.06 ± 16.5	58.76 ± 17.86	57.53 ± 16.18		56.7 ± 16.7	57.2 ± 16.9		
Sex (male/female)	40/50	23/27	18/18		81/95	81/95		
Death (%)	1 (1.1%)	1 (2%)	13 (36.1%)		15 (8.5%)	—		
ANAs (%)	16 (17.7%)	7 (14%)	5 (13.8%)	.728	27 (15.3%)	1 (0.6%)	<.0001****	40
Anti‐dsDNA (%)	8 (8.8%)	4 (8%)	3 (8.3%)	.799	17 (9.7%)	1 (0.6%)	<.0001****	25
Anti‐CCP (%)	5 (5.5%)	11 (22%)	1 (2.7%)	.002**	17 (9.6%)	2 (1.1%)	<.0001****	30
Antiphospholipid (IgG) (%)	0 (0%)	1 (2%)	2 (5.5%)	.092	3 (1.7%)	2 (1.1%)	.500	10
c‐ANCA (%)	2 (2.2%)	0 (0%)	3 (8.3%)	.063	5 (2.8%)	0 (0%)	.030*	10
p‐ANCA (%)	5 (5.5%)	3 (6%)	4 (11.1%)	.516	12 (6.8%)	1 (0.6%)	<.001***	10

Abbreviations: ANAs, anti‐nuclear antibodies; anti‐CCP, anti‐cyclic citrullinated peptide; anti‐dsDNA, anti‐double‐stranded DNA; c‐ANCA, cytoplasmic‐anti neutrophil cytoplasmic antibody; IgG, immunoglobulin G; p‐ANCA, perinuclear‐anti neutrophil cytoplasmic antibody; SEM, standard error of the mean.

*p < .05

**p < .01

***p < .001

****p < .0001.

### 2.2 Enzyme‐linked immunosorbent assay (ELISA) tests

After taking a blood sample from participants, using a centrifuge at 4000 rpm for 5 min, serum samples were instantly separated and stored at −70°C until use. All serum autoantibodies were measured by indirect sandwich ELISA test according to the manufacturer's protocols using an ELISA plate reader (ELISA reader STAT FAX2100). The levels of anti‐CCP, antiphospholipid (IgG), c‐ANCA, and p‐ANCA were analyzed using a sandwich ELISA kit, including Serazym Anti‐CCP IgG (Lot No: 35‐06‐21), Serazym Antiphospholipid screen IgM/IgG (Lot No: 39‐01‐21), Serazym Anti‐PR3 (c‐ANCA) IgG (Lot No: 36‐06‐21), and Serazym Anti‐MPO (p‐ANCA) IgG (Lot No: 35‐06‐21), respectively, according to the manufacturer's instructions (Seramun Company). Besides that, anti‐dsDNA and ANA concentrations were measured with the sandwich ELISA kit, including IMTEC‐dsDNA Antibodies (Lot No: 21004) and IMTEC‐ANA screen (Lot No: 21005) respectively based on the manufacturer's recommendations (Human Company).

### 2.3 Statistical analysis

To describe quantitative variables, mean, and standard error of the mean (SEM) and also for qualitative variables, frequency, and percentage were used. After assessing data normality with a one‐sample Kolmogorov–Smirnov (one‐sample K–S) test, the *χ* ^2^ test was used to compare between patient and control groups. Correlation analysis between variables was also performed using Spearman rank correlation. Data analysis was done using SPSS software version 24.0 (SPSS). The significance level of all tests was statistically considered with *p* values < .05.

## 3 RESULTS

### 3.1 Clinical characteristics of the studied participants

Although there was no meaningful difference between patients and controls in terms of antiphospholipid (IgG), the occurrence of ANAs, anti‐dsDNA, anti‐CCP, c‐ANCA, and p‐ANCA were significantly higher in the COVID‐19 patients compared to serum obtained from healthy subjects (*p* < .0001, *p* < .0001, *p* < .0001, *p* = .03, and *p* < .001, respectively) (Table 1). In addition, a remarkable difference in the number of positive anti‐CCP tests was found, between three subgroups of patients (moderate, severe, and critical) (*p* = .002) with the positive number of anti‐CCP tests increasing significantly in the severe form of COVID‐19.

### 3.2 Correlation analysis between variables

The provided data showed a weak positive correlation between aging and the levels of ANAs and anti‐dsDNA in the patient's group depicted in Table 2 (*r* = .282, *p* = .0001 and *r* = .202, *p* = .007, respectively).

**Table 2:** Correlation between serum autoantibodies and aging.

Age	Patients		Controls	
	Coefficient (*r*)	*p* Value	Coefficient (*r*)	*p* Value
ANAs	.282	.0001	.039	.599
Anti‐dsDNA	.202	.007	.017	.821
Anti‐CCP	−.029	.693	−.048	.525
Antiphospholipid (IgG)	.061	.417	−.018	.802
c‐ANCA	.065	.389	−.022	.769
p‐ANCA	−.002	.978	−.015	.059

Abbreviations: ANAs, anti‐nuclear antibodies; anti‐CCP, anti‐cyclic citrullinated peptide; anti‐dsDNA, anti‐double‐stranded DNA; c‐ANCA, cytoplasmic‐anti neutrophil cytoplasmic antibody; IgG, immunoglobulin G; p‐ANCA, perinuclear‐anti neutrophil cytoplasmic antibody.

## 4 DISCUSSION

The current study found a significant occurrence of autoantibodies (AABs) related to systemic autoimmune diseases, including ANAs, anti‐dsDNA, anti‐CCP, c‐ANCA, and p‐ANCA in COVID‐19 patients. Antinuclear antibodies (ANAs) are a diverse group of AABs that are associated with various systemic autoimmune rheumatic diseases (SARDs), including SLE, sjögren's disease, mixed connective tissue disease (MCTD), systemic sclerosis (SSc), polymyositis, and dermatomyositis. ANAs also become positive in patients with autoimmune hepatitis (AIH).^15^, ^16^

The first study reported the occurrence of ANAs in 50% of the severe and critical forms of COVID‐19 was conducted by Zhou et al.^17^ Although the positive cases of ANAs test were significantly higher compared with serum from the healthy subjects before the COVID‐19 pandemic, in our investigation, there were no remarkable differences in the number of positive ANAs tests between moderate, severe, and critical COVID‐19 patients. Compared to the study performed by Zhou et al., we investigated a larger sample size for the development of ANAs.^17^ In the other investigation, using indirect immunofluorescence assay, Chang et al. reported the presence of ANAs in hospitalized COVID‐19 patients at different serum dilutions with heterogenous patterns, including diffuse, speckled, and nucleolar.^9^ In contrast to our study, Anaya et al.^8^ and Woodruff et al.^7^ found that patients with positive ANAs test were more prone to develop critical illness. This different result can be attributed to various factors affecting ANA development, including aging, sex, history of occupational exposure, drugs, and genetic background such as HLA alleles and viral factors, as well as SARS‐CoV‐2 variants. The mechanisms underlying the development of ANAs in COVID‐19 are similar to SLE and are associated with the activation of TLR7, TLR9, and B cells in the extrafollicular pathway.^18^ The association of ANAs in COVID‐19 patients with disease severity and the long COVID‐19, one of the most important sequelae of COVID‐19, further highlights the importance of ANAs in COVID‐19.^12^, ^19^ A recent study by Son et al. showed that ANA remains positive at 12 months post‐COVID and is associated with post‐acute sequelae of COVID‐19 (PASC) and inflammation.^20^

Considering that aging critically affects COVID‐19 pathogenesis, we assessed the correlation between the prevalence of AABs and aging in COVID‐19 patients.^21^ Our findings showed a weak positive correlation between ANAs and aging in these patients during the COVID‐19 pandemic. Although the age‐related increase in ANAs has been documented in various studies in the healthy population, the remarkable association between positive ANAs test and aging in COVID‐19 patients reported in our study showed that COVID‐19 may accelerate the development of ANAs in the elderly, which reflects the extent of immune dysregulation in senescent COVID‐19 population.^22^, ^23^, ^24^

Anti‐dsDNA antibody was the other SLE‐related autoantibody that significantly became positive in our hospitalized COVID‐19 patients. The development of anti‐DNA autoantibody has been reported by Claudia Gomes and her colleagues previously, which had an association with COVID‐19 severity.^25^ The occurrence of transient lupus‐like manifestations in COVID‐19 patients may prove the importance of ANAs and anti‐dsDNA in these patients.^26^

c‐ANCA and p‐ANCA were two other AABs that significantly became positive in our study. The antineutrophil cytoplasmic antibody (ANCA)‐associated vasculitides (AAVs) are a group of small vessels vasculitis that is recognized by the presence of autoantibodies against the neutrophil's proteins, including proteinase‐3 (c‐ANCA), and myeloperoxidase (p‐ANCA).^27^ The breakdown of tolerance to neutrophil proteins is one of the principal features of AAVs, including granulomatosis with polyangiitis (GPA), microscopic polyangiitis (MPA), and eosinophilic GPA (EGPA).^27^ The development of AAVs following COVID‐19 infection has been documented in the previous investigation.^28^ Dysregulated neutrophil activation in hospitalized COVID‐19 patients may underpin the pathological processes involved in antineutrophil antibody development in COVID‐19 patients.^29^

In the study conducted by Zuo et al., it was found that higher titers of aPL antibodies are associated with neutrophil hyperactivity, comprising the release of neutrophil extracellular traps (NETs), higher platelet counts, more severe respiratory illness, and lower clinical estimated glomerular filtration rate. In contrast to this study that reported a significant occurrence of aPL antibodies in critically ill patients with COVID‐19,^11^ we have just detected IgG aPL autoantibodies in two critically infected COVID‐19 patients. One of the explanations for this discrepancy is the transient presence of aPL antibodies in COVID‐19 patients, which may also occur in our studied population.^30^

COVID‐19 patients displayed remarkable enhancement in autoantibody reactivities, in comparison with the uninfected individuals, and indicated a high prevalence of AABs against immunoregulatory proteins, comprising cytokines, chemokines, complement components, and cell‐surface proteins. Moreover, it was shown that these AABs disturb immune function and perturb virological control by restraining immunoreceptor signaling and changing the composition of peripheral immune cells. Also, murine surrogates of these AABs enhanced illness severity in a murine model of SARS‐CoV‐2 infection.^10^ The significant occurrence of anti‐CCP autoantibodies in hospitalized COVID‐19 patients was one of the unique findings in our investigation. The positive cases of anti‐CCP autoantibody were significantly higher in severe COVID‐19 compared to moderately affected patients. The previous study by Holger Lingel and his colleagues documented elevated levels of anti‐CCP in convalescent patients with COVID‐19.^31^ In contrast to our study, the studies conducted by Anaya et al.^8^ and Woodruff et al.^7^ did not report a significant correlation between the occurrence of anti‐CCP autoantibodies and disease severity. The development of anti‐CCP antibodies depends on various genetic factors, including shared epitope (SE), the alleles of HLA‐DRB1, and polymorphism in peptidyl arginine deiminase 4 (PADI4), as well as environmental factors such as smoking and infection with *Porphyromonas gingivalis*, the agent of periodontitis which may also affect the development of anti‐CCP in COVID‐19 patients with different disease severity.^32^, ^33^, ^34^, ^35^ A case of the onset of RA following COVID‐19 has been reported by Rashmi Roongta et al. previously.^36^ The COVID‐19 patients were moreover indicated to have significantly higher risks of developing several autoimmune disorders, including RA.^37^ Similarly, in a cohort study, it was revealed that COVID‐19 was associated with an enhanced risk of being newly diagnosed with autoimmune disease 3–15 months following SARS‐CoV‐2 infection. Also, COVID‐19 depicted the strongest relationship with vascular autoimmune diseases.^38^ However, in another study, no association was found between COVID‐19 illness and subsequent development of RA‐related autoimmunity, nor signs or symptoms of RA in at‐risk people.^39^ The association between local NETs and the development of anti‐CCP has been described in people at risk of RA development.^40^ Activated neutrophils act to release DNA in the process of NETosis. NETs then display citrullinated antigens that are thought to contribute to the development of anti‐CCP.^41^ SARS‐CoV‐2 infection is a potent inducer of NETosis and citrullination, which leads to neoantigens that trigger the development of anti‐CCP.^3^ The previous studies showed the association between the extent of NETosis and the COVID‐19 severity.^42^, ^43^, ^44^ Since the role of NETs has been documented in the development of anti‐CCP, the higher production of anti‐CCP in severe forms of COVID‐19 patients in our study may be attributed to the extent of NETosis in severe forms of COVID‐19, which warrants further investigations. In contrast, we could not find higher levels of anti‐CCP in our critical patients, which may be attributed to the effects of other factors that provoke the development of anti‐CCP, including shared epitope (SE), PADI4 polymorphism, smoking status, and other genetic and environmental factors that affect the production of anti‐CCP.^32^, ^33^ The anti‐CCP can be detected in a median of 4.5 years before developing the RA phenotype.^2^ In line with our findings, a recent study found that COVID‐19 patients indicated a higher frequency of IgG anticyclic citrullinated peptide third‐generation (CCP3) antibodies than pre‐pandemic controls. In this study, hospitalized patients with COVID‐19 displayed latent rheumatic and antiphospholipid autoimmunity. Moreover, ANAs, RF, CCP3, and aPLs were reported to be the most common autoantibodies in COVID‐19 patients. Also, the presence of ANAs and RF antibodies was considered as a risk factor for critical illness.^8^ The occurrence of anti‐CCP in COVID‐19 patients may predict the possible development of RA in the COVID‐19 population with a predisposing genetic background, including patients with specific HLA‐DRB1 alleles (shared epitope).

## 5 CONCLUSION

In conclusion, our study documented the development of AABs associated with various systemic autoimmune rheumatic diseases in hospitalized COVID‐19 patients. To the best of our knowledge, this is the first study with a large sample size that reported the occurrence of anti‐CCP in a severe form of COVID‐19.

## AUTHOR CONTRIBUTIONS

**Mahdi Taghadosi**: Design of the research study; data collection; supervision; writing the manuscript and revising it. **Seyed Askar Roghani, Mohammad Dastbaz, and Ramin Lotfi**: Data collection and helped with writing the manuscript and revising it. **Afsaneh Shamsi**: Helped with writing the manuscript and language editing. **Zahra Abdan, Rezvan Rostampour, and Bijan Soleymani**: Helped with writing the manuscript and revised it. **Mohammad Hossein Zamanian, Parviz Soufivand, and Mehran Pournazari**: Specialist physicians referred the COVID‐19 patients for sampling. All authors reviewed and approved the final version of the manuscript for publication.

## CONFLICT OF INTEREST STATEMENT

The authors declare no conflict of interest.

## ETHICS STATEMENT

This study was performed in line with the principles of the Declaration of Helsinki. Approval was granted by the Ethics Committee of the Kermanshah University of Medical Sciences (KUMS), Kermanshah, Iran (Ethical code: IR.KUMS.MED.REC.1400.058). Informed consent was obtained from all individual participants included in the study. The authors affirm that human research participants provided informed consent for the publication of the manuscript results.

## Data Availability

The data sets generated during and/or analyzed during the current study are available from the corresponding author upon reasonable request.
